# Suspected Pembrolizumab-Associated Perinuclear Antineutrophil Cytoplasmic Antibody (p-ANCA) Vasculitis With Progressive Kidney Injury in a Patient With Metastatic Renal Cell Carcinoma and Limited Renal Reserve: A Diagnostic Challenge

**DOI:** 10.7759/cureus.115080

**Published:** 2026-08-24

**Authors:** Ray Goshtaseb, Ying Luu, Kristine Sarmosyan, Lidia Lopez, Alexandra Drakaki

**Affiliations:** 1 Nephrology, University of California, Los Angeles, Los Angeles, USA; 2 Nephrology, Ronald Reagan University of California Los Angeles Medical Center, Los Angeles, USA; 3 Nephrology, Ronald Reagan Medical Center, Los Angeles, USA; 4 Hematology-Oncology, University of California, Los Angeles, Los Angeles, USA

**Keywords:** anca-associated glomerulonephritis (anca-gn), anca-associated vasculitis, immune checkpoint inhibitor, immune-related adverse event, p-anca positive vasculitis, pembrolizumab, proteinuria

## Abstract

Immune checkpoint inhibitors (ICIs) have significantly improved outcomes for patients with advanced malignancies but can result in immune-related adverse events involving multiple organ systems. Renal immune-related adverse events most commonly present as acute tubulointerstitial nephritis; however, immune checkpoint inhibitor-associated glomerular diseases, including antineutrophil cytoplasmic antibody (ANCA)-associated vasculitis, are rare but potentially severe complications. We report the case of a patient with metastatic clear cell renal cell carcinoma treated with pembrolizumab who developed progressive renal abnormalities, including worsening proteinuria, rising serum creatinine, and newly detected high-titer perinuclear ANCA (p-ANCA) positivity. The patient underwent right nephrectomy in 2019 and subsequent left partial nephrectomy in 2024, resulting in limited renal reserve. Prior to initiation of adjuvant pembrolizumab in April 2024, his serum creatinine was 1.18 mg/dL without significant proteinuria. During subsequent pembrolizumab therapy, his creatinine increased to 1.6 mg/dL and urine protein-to-creatinine ratio increased from 0.2 g/g to 1.2 g/g. Serologic evaluation demonstrated p-ANCA positivity at a titer of 1:640 with negative cytoplasmic antineutrophil cytoplasmic antibody (c-ANCA) (<1:20), raising concern for an immune checkpoint inhibitor-associated vasculitic process. Kidney biopsy, the diagnostic gold standard for pauci-immune glomerulonephritis, was deferred due to concern for complications in his solitary partially functioning kidney. Based on the temporal association with pembrolizumab exposure, renal findings, ANCA serology, pulmonary abnormalities, and exclusion of alternative causes, probable pembrolizumab-associated ANCA vasculitis was considered. This case highlights the diagnostic challenges of immune checkpoint inhibitor-associated vasculitis when tissue confirmation cannot safely be obtained and emphasizes the importance of early recognition and multidisciplinary management.

## Introduction

Immune checkpoint inhibitors targeting programmed cell death protein 1 (PD-1) have transformed the treatment landscape for multiple malignancies, including metastatic renal cell carcinoma. Pembrolizumab enhances antitumor immunity by promoting T-cell activation against malignant cells; however, this immune activation may also lead to autoimmune toxicities affecting multiple organ systems. These immune-related adverse events can affect the endocrine system, gastrointestinal tract, lungs, skin, nervous system, and kidneys [1].

Renal complications associated with immune checkpoint inhibitors are uncommon but clinically important. The most frequently reported kidney lesion is acute tubulointerstitial nephritis, although a spectrum of immune-mediated glomerular diseases has been described, including minimal change disease, membranous nephropathy, IgA nephropathy, and pauci-immune glomerulonephritis associated with antineutrophil cytoplasmic antibody (ANCA) vasculitis [2-4].

ANCA-associated vasculitis is characterized by necrotizing inflammation of small vessels and may involve both the kidneys and lungs. Renal involvement typically manifests as rapidly progressive glomerulonephritis, and kidney biopsy demonstrating pauci-immune necrotizing and crescentic glomerulonephritis remains the diagnostic gold standard [5].

However, kidney biopsy may not always be feasible, particularly in patients with solitary kidneys, reduced renal reserve, or increased procedural risk. As immune checkpoint inhibitors become more widely used in oncology, clinicians may encounter patients with suspected immune-mediated renal disease where definitive histologic confirmation cannot safely be obtained.

We present a case of probable pembrolizumab-associated perinuclear antineutrophil cytoplasmic antibody (p-ANCA) vasculitis in a patient with metastatic clear cell renal cell carcinoma who developed progressive kidney dysfunction and proteinuria after initiation of therapy. Renal biopsy was deferred because of previous bilateral renal surgery and concern for irreversible loss of remaining kidney function.

## Case presentation

We present a case of a 68-year-old male patient with a medical history significant for metastatic clear cell renal cell carcinoma (ccRCC), recurrent pneumonia, type 2 diabetes mellitus, hypertension, and secondary adrenal insufficiency who was referred to nephrology for evaluation and management of kidney disease and worsening proteinuria.

He was diagnosed with bilateral clear cell renal cell carcinoma in September 2019 with metastatic disease. patient underwent right radical nephrectomy in September 2019. During surveillance imaging, a left renal lesion was identified. The patient underwent left partial nephrectomy at UCLA in February 2024. Intraoperative findings demonstrated renal vein involvement, resulting in pathologic upstaging to Stage III disease.

Following left partial nephrectomy, the patient was started on adjuvant pembrolizumab (Keytruda) in April 2024. At the initiation of pembrolizumab therapy, his renal function was stable and without any significant baseline proteinuria. Following bilateral renal surgery, the patient had limited renal reserve due to a right nephrectomy and left partial nephrectomy. While receiving pembrolizumab therapy, his renal function started worsening (Table 1).

**Table 1:** Laboratory results before and after pembrolizumab initiation. Laboratory results showing worsening of proteinuria, creatinine, and p-ANCA association after pembrolizumab initiation. p-ANCA: perinuclear antineutrophil cytoplasmic antibody; c-ANCA: cytoplasmic antineutrophil cytoplasmic antibody.

Laboratory parameter	Before pembrolizumab	After pembrolizumab initiation	Reference range
Serum creatinine (mg/dL)	1.18	1.60	0.60-1.30 (mg/dL)
Urine protein-to-creatinine ratio (g/g)	0.20	1.20	<0.20 (g/g)
p-ANCA	Not available	Positive (1:640)	Negative
c-ANCA	Not available	Negative (<1:20)	Negative
Proteinuria (g/g)	0.2	1.2	0-0.2 g/g

His serum creatinine increased from the baseline after pembrolizumab initiation. In parallel, urinary protein excretion also increased significantly, representing a substantial increase compared with the previous baseline (Table 1).

The patient had a history of well-controlled diabetes mellitus, which could contribute to chronic kidney disease and baseline proteinuria. However, the timing and magnitude of worsening proteinuria, along with new autoimmune findings and pulmonary abnormalities, raised concern for a superimposed immune-mediated renal process.

During the same period, he developed recurrent pulmonary abnormalities and repeated episodes initially treated as pneumonia. Given his ongoing pembrolizumab exposure, worsening kidney function, increasing proteinuria, and pulmonary findings, evaluation for systemic vasculitis was pursued.

Autoimmune evaluation revealed p-ANCA positivity, and c-ANCA was negative. The high-titer p-ANCA positivity raised concern for a possible ANCA-associated vasculitic process (Table 1).

The patient was evaluated by a multidisciplinary team including nephrology, rheumatology, pulmonology, and oncology. The patient's medication history was reviewed carefully. Hydralazine, a known trigger of drug-induced ANCA-associated vasculitis, had previously been discontinued, making hydralazine-related disease less likely. The temporal relationship between pembrolizumab initiation and subsequent development of renal and autoimmune abnormalities raised concern for immune checkpoint inhibitor-associated vasculitis.

Kidney biopsy was considered to establish a definitive diagnosis. However, the patient had undergone right nephrectomy and left partial nephrectomy, leaving a solitary partially functioning kidney.

Given the increased procedural risks, including bleeding, loss of residual renal function, and potential progression to dialysis dependence, the decision was made not to proceed with renal biopsy.

Although histologic confirmation of pauci-immune necrotizing glomerulonephritis could not be obtained, the combination of recent pembrolizumab exposure, normal renal function and minimal proteinuria before therapy, progressive increase in creatinine, with simultaneous increase in urine protein-to-creatinine ratio (UPCR), high-titer p-ANCA positivity, pulmonary abnormalities, and lack of another unifying diagnosis was considered highly concerning for probable pembrolizumab-associated ANCA vasculitis.

## Discussion

Immune checkpoint inhibitors have become an integral component of cancer treatment; however, their immune-enhancing effects can lead to autoimmune complications involving multiple organ systems. Renal immune-related adverse events are relatively uncommon but may result in significant morbidity if not recognized promptly. Acute tubulointerstitial nephritis remains the most commonly reported renal toxicity associated with immune checkpoint inhibitors, while ANCA-associated vasculitis remains a rare but clinically important complication [1-4].

Several cases of immune checkpoint inhibitor-associated ANCA vasculitis have been reported in the literature, including cases involving pembrolizumab and other PD-1 inhibitors. Most published cases demonstrated positive myeloperoxidase (MPO)-ANCA or proteinase 3 (PR3)-ANCA serology with kidney biopsy findings consistent with pauci-immune necrotizing crescentic glomerulonephritis [5-8].

The current case is unique because the clinical diagnosis had to be made without renal biopsy confirmation. The patient had a particularly high-risk profile for biopsy-related complications because of prior right nephrectomy and left partial nephrectomy, leaving limited remaining renal tissue. In such circumstances, the risk of biopsy-associated permanent renal injury must be carefully weighed against the diagnostic benefit.

The temporal relationship between pembrolizumab exposure and development of renal abnormalities supports a possible immune checkpoint inhibitor-mediated mechanism. Importantly, before starting adjuvant pembrolizumab in April 2024, the patient had stable renal function, with a creatinine of 1.18 mg/dL and no significant proteinuria. The subsequent development of progressive kidney dysfunction, increasing proteinuria, and high-titer p-ANCA positivity after treatment exposure suggests a possible pembrolizumab-triggered autoimmune process.

The mechanisms underlying ICI-associated ANCA-associated vasculitis remain incompletely understood, but blockade of programmed cell death protein 1 (PD-1) and programmed death-ligand 1 (PD-L1) provides a plausible explanation. PD-1 normally maintains peripheral immune tolerance by limiting autoreactive T-cell activation; its inhibition by pembrolizumab may therefore promote autoreactive T- and B-cell responses. This can lead to the production of MPO- or PR3-ANCAs, which activate primed neutrophils, causing degranulation and endothelial injury. Neutrophil extracellular traps (NETs) further amplify inflammation by exposing MPO and PR3 as autoantigens, sustaining ANCA production. Complement activation, particularly C5a, additionally primes neutrophils and enhances vascular injury. Together, these mechanisms suggest that PD-1 blockade may trigger or unmask ANCA-associated vasculitis through loss of immune tolerance and amplification of neutrophil-driven inflammation, although direct causal evidence remains limited. Clinical reports of de novo or relapsing ANCA-associated vasculitis after PD-1 inhibitors support this association [9-13].

Alternative causes of kidney dysfunction were considered. The patient had diabetes mellitus, but it was well controlled, which could not contribute to chronic kidney disease and proteinuria; however, diabetic nephropathy alone would not fully explain the relatively rapid increase in proteinuria, new high-titer p-ANCA positivity, and pulmonary abnormalities. Hydralazine-associated ANCA vasculitis was also considered but was less likely because the medication had already been discontinued.

Management of suspected immune checkpoint inhibitor-associated vasculitis generally includes discontinuation of the suspected offending agent and consideration of immunosuppressive therapy depending on severity and organ involvement. Corticosteroids are commonly used as initial treatment, while rituximab or cyclophosphamide may be considered for organ-threatening disease following principles used for primary ANCA-associated vasculitis [7-11].

In this patient, discontinuation of pembrolizumab is being considered if clinical suspicion remains high and if kidney function continues to decline or proteinuria continues to increase; immunosuppressive therapy will be considered after discussion among the nephrology, rheumatology, pulmonology, and oncology teams.

We acknowledge the limitation of incomplete longitudinal clinical and serologic follow-up after pembrolizumab discontinuation. Extended follow-up with serial serum creatinine, proteinuria, and ANCA measurements would be valuable to better characterize the patient's long-term response. Such data may help further clarify the natural history and pathophysiology of immune checkpoint inhibitor-associated ANCA vasculitis.

The decision to initiate immunosuppression must carefully balance potential renal benefit against significant infectious risk. This patient has multiple factors increasing susceptibility to infection, including recurrent pneumonia, malignancy, chronic corticosteroid therapy for adrenal insufficiency, and prior immune checkpoint inhibitor exposure.

This case emphasizes that clinicians must maintain awareness of rare immune checkpoint inhibitor-associated vasculitic complications and recognize that diagnosis may occasionally rely on clinical judgment when biopsy is unsafe.

## Conclusions

Pembrolizumab-associated ANCA vasculitis is a rare but potentially serious immune-related adverse event. This case highlights the diagnostic challenge of suspected immune checkpoint inhibitor-associated vasculitis in a patient with limited renal reserve after bilateral renal surgery. Although kidney biopsy was deferred because of the risk to the patient's solitary partially functioning kidney, the temporal relationship to pembrolizumab therapy, progressive kidney dysfunction, worsening proteinuria, high-titer p-ANCA positivity, and pulmonary abnormalities strongly supported a probable pembrolizumab-associated vasculitic process.

When a kidney biopsy is contraindicated, integration of clinical findings, serologic testing, medication history, imaging, and multidisciplinary evaluation is essential for diagnosis and management. Early recognition, discontinuation of the suspected offending agent, and consideration of immunosuppressive therapy when appropriate may help prevent irreversible renal and pulmonary injury.
